# Self-Administration of Toluene Vapor in Rats

**DOI:** 10.3389/fnins.2020.00880

**Published:** 2020-08-18

**Authors:** Kevin M. Braunscheidel, Wesley N. Wayman, Michael P. Okas, John J. Woodward

**Affiliations:** Department of Neuroscience, The Medical University of South Carolina, Charleston, SC, United States

**Keywords:** addiction, inhalants, vapor self-administration, drug abuse, drug-seeking, reinstatement

## Abstract

Inhalants, including volatile organic solvents such as toluene, continue to be one of the most prevalent, and often first substances abused by adolescents. Like other drugs of abuse, toluene affects the function of neurons within key brain reward circuits including the prefrontal cortex, ventral tegmental area, and nucleus accumbens. However, preclinical models used to study these toluene-induced adaptations generally employ passive exposure paradigms that do not mirror voluntary patterns of solvent exposure observed in humans. To address this shortcoming, we developed an inhalation chamber containing active and inactive nose pokes, cue lights, flow-through vaporizers, and software-controlled valves to test the hypothesis that rats will voluntarily self-administer toluene vapor. Following habituation and self-administration (SA) training rats achieve vapor concentrations associated with rewarding effects of toluene, and maintain responding for toluene vapor, but not for air. During extinction trials, rats showed an initial burst of drug-seeking behavior similar to that of other addictive drugs and then reduced responding to Air SA levels. Responding on the active nose poke recovered during cue-induced reinstatement but not following a single passive exposure to toluene vapor. The results from these studies establish a viable toluene SA protocol that will be useful in assessing toluene-induced changes in addiction neurocircuitry.

## Introduction

Volatile organic solvents including toluene have important commercial uses and are found in many common household and commercial products (e.g., spray paints, cleaners, adhesives). When voluntarily inhaled at high concentrations in humans, they produce intoxication and euphoria (Balster et al., 2009; Gigengack, 2014; Camara-Lemarroy et al., 2015). Inhalants are often one of the first drugs of abuse tried among adolescents and young adults likely reflecting their ease of access and relatively low cost. The National Institute on Drug Abuse estimates that roughly 21.7 million Americans aged 12 and older have used inhalants at least once in their lives (Substance Abuse and Mental Health Services Administration, 2013), a rate that may be on the rise (Johnston et al., 2018). Despite this prevalence, inhalants remain an understudied class of abused substances with only a handful of NIH funded grants devoted to their study (Woodward and Beckley, 2014).

Research conducted over the past several years shows that toluene and other inhalants alter the structure and function of neurons within key brain regions (e.g., prefrontal cortex, nucleus accumbens, ventral tegmental area) of the reward circuitry (Beckley et al., 2013; Braunscheidel et al., 2017; Wayman and Woodward, 2017, 2018; Wu et al., 2018; Cruz et al., 2019). Although these neurophysiological alterations support the idea that that toluene’s addictive properties result from actions on reward pathways, the majority of preclinical studies that have examined the rewarding properties of toluene used passive exposure paradigms that do not mirror human inhalant abuse (Funada et al., 2002; Gerasimov et al., 2003; Lee et al., 2006; Wayman and Woodward, 2018; Wu et al., 2018).

The gold-standard for modeling addiction in the preclinical setting has been operant-based self-administration; for review, see Sanchis-Segura and Spanagel (2006). In this paradigm, rodents or non-human primates self-administer a drug (orally or intravenously via a chronically implanted catheter) for multiple hours per day over the course of several weeks. This is followed by various drug-cue extinction, reinstatement or relapse trials.

The development of a rodent model of inhalant self-administration has lagged behind other drugs of abuse presumably due to the difficulty in controlling inhalant concentrations and overcoming initial aversive effects associated with solvent odor. In the first of two published attempts to model toluene abuse, Weiss et al. (1979) trained four squirrel monkeys outfitted with a custom inhalation helmet to lever-press for a toluene vapor. This approach required a significant investment of time and economic resources and has not been repeated. The second study involved a single, 30 min intravenous administration of toluene in mice (Blokhina et al., 2004) and to our knowledge has not been repeated. This approach is not optimal for using rodent models to study inhalant abuse for several reasons: (1) it does not mimic the inhaled route of administration in humans, (2) as a solvent, toluene can induce significant vein damage (Kulkarni et al., 2015), and (3) it prevents the study of critical aspects of addiction that require multiple administration sessions. These include measures such as acquisition, drug use escalation, withdrawal, extinction, drug-seeking, drug-craving, and relapse among others.

To address these shortcomings, we generated a low-cost inhalation self-administration chamber using readily available Med Associates (Saint Albans City, VT, United States) hardware and software. We then carried out initial studies to determine the feasibility of using this system to train rats to voluntarily self-administer toluene vapor.

## Materials and Methods

### Animals

Twenty male Sprague-Dawley rats (Envigo RMS, Indianapolis, IN, United States) arrived at the MUSC temperature/humidity-controlled vivarium at age P21 and were housed in pairs under a 12 h light/dark cycle. Food and water were available *ad libitum*. Rats were used in accordance with protocols approved by the Medical University of South Carolina Institutional Animal Care and Use Committee. Pairs of adolescent (P21) rats were first randomly assigned to self-administer air (Air SA; *n* = 6) or toluene (Tol SA; *n* = 14) and allowed to habituate to their home cage.

### Vapor Chamber

**FIGURE 1:**
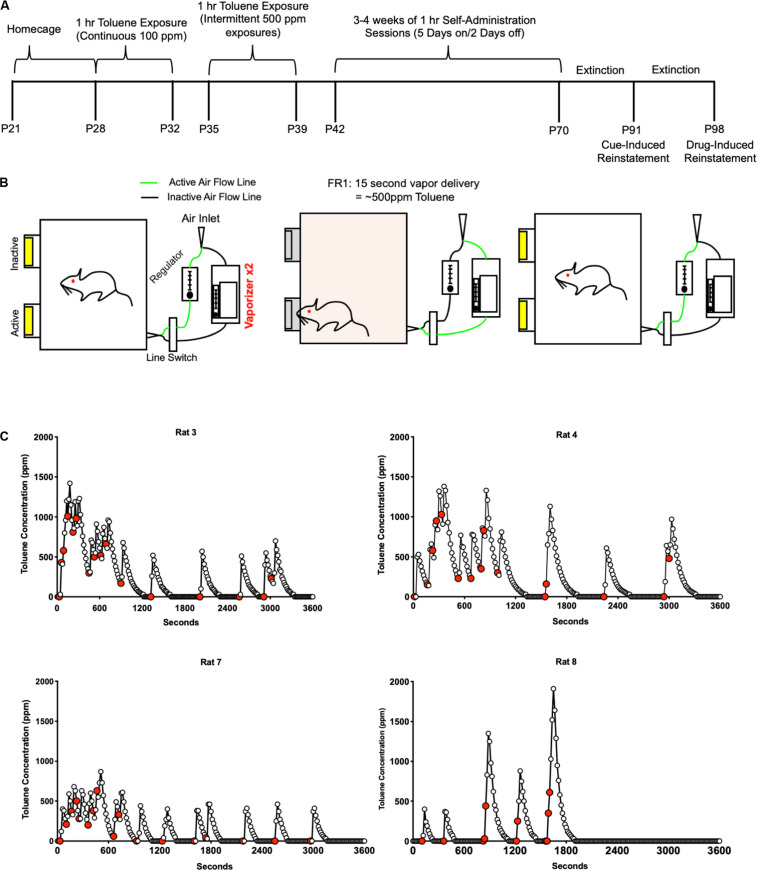
Toluene self-administration protocol. **(A)** Timeline for acquisition, extinction, and reinstatement of operant responding for toluene vapor. **(B)** Illustration of the vapor SA apparatus. Active nose pokes triggered a 15 s exposure of air or toluene vapor while inactive nose pokes had no consequence. Additional responses during the 15 s drug delivery had no additional consequence. For Air SA and extinction sessions, the toluene vaporizers were replaced with a second air regulator. **(C)** Graphs show examples of 1 h self-administration sessions with toluene vapor concentrations measured every 15 s. Red filled symbols represent times of active vapor deliveries.

The self-administration apparatus consisted of a 30 cm × 30 cm × 30 cm anesthesia chamber (Plas Labs, Lansing, MI, United States) fitted with a vapor inlet and outlet and modified to accommodate two lighted nose pokes (Med Associates, Saint Albans City, VT, United States). The nose pokes were spaced 17 cm apart and secured onto a 24 cm × 29 cm pegboard fitted to the inner dimensions of the chamber, creating an inner wall. Toluene (Sigma-Aldrich, St. Louis, MO, United States) was delivered to the chamber via two sevoflurane vaporizers and air- and toluene-flow were regulated via solenoid equipped valves (Med Associates, Saint Albans City, VT, United States) controlled by MED-PC IV software (Med Associates, Saint Albans City, VT, United States). Vapor concentrations within the chamber were recorded during training and self-administration with a portable toluene gas detector (XP-3160, DOD Technologies, Cary, IL, United States) that uses a flow-through design giving a continuous readout of toluene concentrations. For the examples shown in Figure 1, readings were recorded every 15 s.

### Toluene Self-Administration Paradigm

Rats used in the current study began training during early adolescence to mimic human consumption patterns. Rats arrived at 21 days of age (P21) and were given 7 days of home cage habituation (Figure 1A). Beginning at P28, rats were placed in the vapor chamber for 60 min each day for five consecutive days during which they were exposed to a constant flow of air or 100 ppm toluene (5 L/min) to acclimate them to the vapor chamber and the toluene odor. After 2 days in their home cage, rats (P35) were exposed to five daily sessions of intermittent exposure to toluene vapor (∼500 ppm) to acclimate them to the “click” sound of solenoids that controlled the delivery of toluene or air to the chamber. During the 1-h toluene sessions, each non-contingent exposure delivered toluene vapor into the chamber for 15 s followed by an air washout for 285 s. This resulted in a concentration of ∼500 ppm for each toluene vapor delivery. This cycle was repeated 12 times with an inter-vapor delivery interval of 5 min. Following 2 days off in their home cage, rats (P42) underwent daily 1 h trials of toluene self-administration. In this phase, rats had access to two nose pokes illuminated with a cue light within the nose poke. Entries into the “inactive” nose poke had no consequence. Entries into the “active” nose poke turned off the cue lights in both nose pokes and activated the solenoids to change the vapor flow from air to toluene for 15 s. During this 15 s period timeout period, additional nose pokes were recorded but had no consequence. After 15 s, the cue lights illuminated, and the solenoids switched back to air. Toluene concentrations in the chamber returned to 0 ppm after approximately 30 s of airflow if no additional nose pokes were made (Figure 1C).

Rats underwent self-administration (SA) sessions for three consecutive weeks (5 days of SA followed by 2 days off in the home cage). A subset then underwent extinction (EXT) sessions for the next 3 weeks. During these sessions, active pokes no longer triggered the conditioned cues or vapor deliveries. Following extinction, cue-induced reinstatement was tested where active nose pokes triggered the conditioned stimulus but with just air delivery. Rats underwent one more week of extinction before testing for drug-induced reinstatement. This test day was a normal extinction session except rats were given a single dose of toluene vapor (∼500 ppm for 15 s) immediately prior to testing. The Air SA rats that served as a control group were treated exactly as described above without toluene vaporizers attached to the apparatus.

### Statistical Analysis

During all self-administration sessions, the number of toluene deliveries, active and inactive nose pokes were recorded. From these data, we calculated active nose poke preference [active/(active + inactive)], toluene delivery success rate (deliveries/active pokes). Latency to first toluene delivery was recorded in a subset of animals. Data were analyzed with one- and two- way repeated measures ANOVAs and Sidak’s or Dunnett’s *post hoc* tests corrected for multiple comparisons using GraphPad Prism Software v8 (GraphPad Software, Inc., San Diego, CA, United States). In some cases, data for the air and toluene groups was averaged and compared using a two-way unpaired *t*-test. In all cases, differences were considered significantly different when *p* < 0.05.

## Results

### Self-Administration

Figure 1 summarizes the timeline of the toluene self-administration (Tol SA) training and testing protocol (Figure 1A) and illustrates the setup of the self-administration chamber apparatus (Figure 1B). Representative examples of toluene vapor concentrations achieved by rats during single 1 h self-administration sessions are shown in Figure 1C and indicate subject-dependent patterns of self-administration.

**FIGURE 2:**
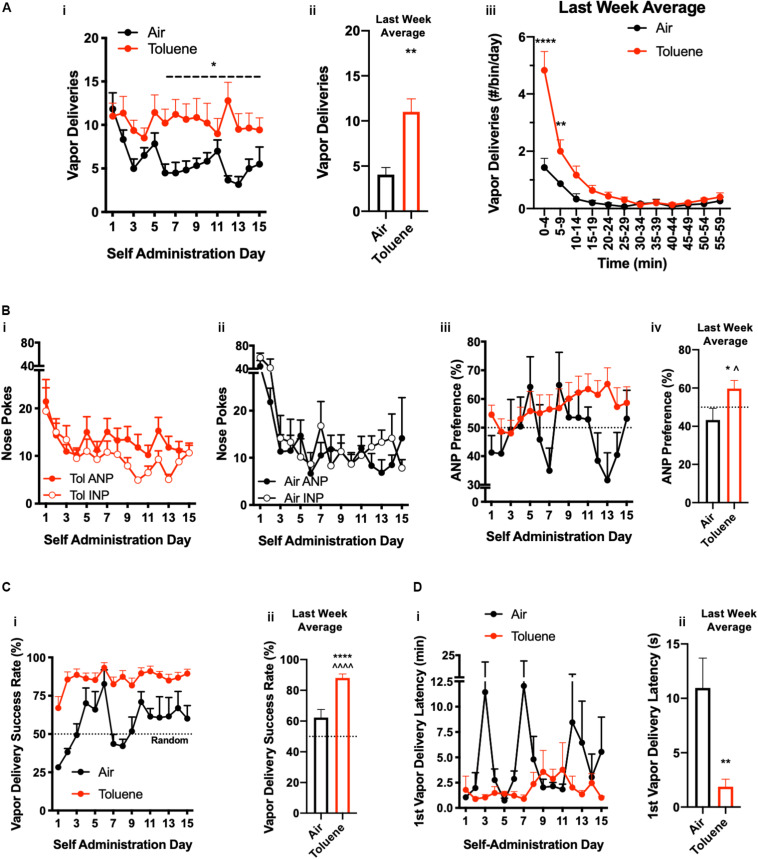
Toluene self-administration. Operant responding for toluene (Tol) vapor or Air across 3 weeks of SA training. **(A-i)** Number of daily vapor deliveries for Air SA and Tol SA rats; ^∗^*p* < 0.05 Tol vs. Air, two-way repeated measures ANOVA. **(A-ii)** Summary graph showing last week average vapor deliveries for Air or Tol rats; ^∗∗^*p* < 0.01, Tol vs. Air, Student’s *t*-test. **(A-iii)** Time course of vapor deliveries during the 1 h session shown in 5 min bins; ^∗∗^*p* < 0.01, ^∗∗∗∗^*p* < 0.0001, Student’s *t*-test, Tol vs. Air. **(B-i)** Number of active nose pokes (ANP) and inactive nose pokes (INP) in Tol SA rats. **(B-ii)** Number of active nose pokes (ANP) and inactive nose pokes (INP) in Air SA rats. (**B-iii)** ANP preference (ANPs/total NPs) across 3 weeks of testing (dotted line denotes no preference). **(B-iv)** Summary graph showing last week average active nose pokes for Air or Tol rats; ^∗^*p* < 0.05, Student’s *t*-test Tol vs. Air; ^*p* < 0.05, one-sample *t*-test Tol vs. 50% ANP preference. **(C-i)** Vapor delivery success rate (vapor deliveries/ANPs) across 3 weeks of testing (dotted line denotes random entry). **(C-ii)** Summary graph showing last week average vapor delivery success rate for Air or Tol rats; ^∗∗∗∗^*p* < 0.0001, Student’s *t*-test Tol vs. Air; ^^^^*p* < 0.0001, one-sample *t*-test Tol vs. 50% vapor delivery success rate. **(D-i)** Latency to first vapor delivery. **(D-ii)** Summary graph showing last week average latency to first vapor delivery for Air or Tol rats; ^∗∗^*p* < 0.05, Student’s *t*-test Tol vs. Air; ^*p* < 0.05 one-sample *t*-test Tol vs. 50% ANP preference. All data are mean ± SEM. Air *N* = 6, Tol *N* = 14 **(A–C)**, 6 **(D)**.

Figure 2 compares the responding of Air and Tol SA groups on the active and inactive nose pokes over 3 weeks of operant self-administration training. Active vapor deliveries were similar between Air SA and Tol SA groups for the first several sessions of SA (Days 1–5) but began to show separation by Day 6 (Figure 2A-i). Analysis of data averaged across the final 2 weeks of testing (Days 6–15) revealed a main effect of toluene on the number of vapor deliveries (two-way repeated measures ANOVA, *F*_(__1_,_18__)_ = 4.44, *p* < 0.0493). During the last week of training, Tol SA rats self-administered significantly more vapor deliveries compared to Air SA controls (Figure 2A-ii, two-way unpaired *t*-test, *t*_(__18__)_ = 3.07, *p* = 0.007). When responding over the 1 h session was broken down into 5-min bins, there was a significant interaction between treatment and time (Figure 2A-iii, two-way repeated measures ANOVA, *F*_(__11_,_120__)_ = 10.97, *p* < 0.0001). *Post hoc* testing revealed that this interaction was driven by increased responding by the Tol SA group in the first 10 min of the SA session (Air vs. Tol: minutes 0–4, *t*_(__120__)_ = 11.71, *p* < 0.0001; minutes 5–9 *t*_(__120__)_ = 3.90, *p* = 0.0019; Sidak’s *post hoc* test).

In order to determine if toluene self-administration was goal-directed or simply reflected a drug-induced increase in responding, we analyzed active nose poke preference, vapor delivery success rate, and latency to first vapor delivery over the course of SA training. In both Tol (Figure 2B-i) and Air (Figure 2B-ii) SA rats, the number of active (ANP) and inactive nose pokes (INP) were initially high but dropped off significantly over the 3 weeks of testing (Two-way ANOVA, main effect of time, Tol SA *F*_(__14_,_362__)_ = 4.96, *p* < 0.001; Air SA *F*_(__14_,_139__)_ = 13.03, *p* < 0.0001). In addition, while the total number of ANPs did not differ from INPs for either group over the entire self-administration period (Two-way ANOVA, main effect of nosepoke, Tol SA *F*_(__1_,_26__)_ = 1.94, *p* = 0.18; Air SA *F*_(__1_,_10__)_ = 1.02, *p* = 0.33), a significant preference for the ANP (Figures 2B-iii,iv) developed in Tol SA (last week average, one- sample *t*-test vs. 50%: *t*_(__13__)_ = 2.29, *p* = 0.039) but not Air SA rats (*t*_(__5__)_ = 1.105, *p* = 0.32). In addition, the ANP preference for Tol SA rats over this time was significantly different from that of the Air SA animals (two-tailed unpaired *t*-test, *t*_(__18__)_ = 2.16, *p* = 0.04). Vapor delivery success rate (vapor deliveries/ANP; Figure 2C-i) was analyzed to determine whether rats learned that responding on the active nose poke during an active vapor delivery had no consequence. Over the entire SA training period, there was a main effect of time (*F*_(__14_,_251__)_ = 6.98, *p* < 0.0001), drug (*F*_(__1_,_18__)_ = 36.39, *p* < 0.0001) and interaction (*F*_(__14_,_251__)_ = 2.32, *p* < 0.005) on vapor delivery success rate. Across the final week of testing, Tol SA, but not Air SA rats had a vapor delivery success rate higher than that predicted by random entry (Figure 2C-ii, one-sample *t*-test vs. 50%, Tol SA, *t*_(__13__)_ = 14.84, *p* < 0.0001; Air SA, *t*_(__5__)_ = 2.34, *p* = 0.0663) and also had a higher vapor delivery success rate compared to Air SA rats (two-tailed unpaired *t*-test, *t*_(__18__)_ = 4.99, *p* < 0.0001). As a measure of motivation to obtain reward, we analyzed the latency to first vapor delivery over the course of SA testing (Figure 2D-i). Over the 3 week self-administration period, there was a main effect of drug (*F*_(1,30)_ = 12.23, *p* = 0.002); time (*F*_(2,30)_ = 3.64, *p* = 0.039) and interaction (*F*_(2,30)_ = 4.16, *p* = 0.025). Consistent with that found when responding was broken into 5 min bins (Figure 2A-iii), Tol SA rats were significantly quicker to respond for their first toluene vapor delivery compared to Air SA controls during the final week of testing (Figure 2D-ii, two-tailed unpaired *t*-test, *t*_(__10__)_ = 3.209, *p* = 0.0093).

### Extinction and Reinstatement

**FIGURE 3:**
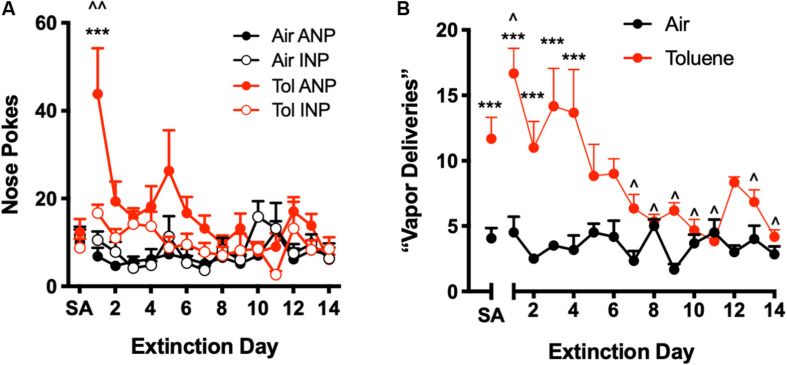
Extinction of operant responding for toluene vapor. During extinction training, a nose poke in the active hole had no consequence (i.e., did not trigger toluene delivery or conditioned cues). **(A)** Number of active (ANP) and inactive nose pokes (INP) for Air SA and Tol SA rats during extinction training. SA is average ANP and INP during last week of self-administration; ^^*p* < 0.01 Tol ANP EXT vs. Tol SA, ^∗∗∗^*p* < 0.001 Tol ANP EXT vs. Tol INP EXT, Sidak’s *post hoc*. **(B)** Number of ANPs that would trigger a toluene vapor delivery under normal SA conditions. SA is average number of vapor deliveries during last week of self-administration; ^*p* < 0.05, Tol EXT vs. Tol SA, ^∗∗∗^*p* < 0.001 Tol vs. Air, Sidak’s *post hoc*. All data are mean + SEM. Air *N* = 6, Tol *N* = 6.

Following SA testing, operant responding was monitored during extinction training (EXT) during which a nose poke in the active hole had no consequence. A spike in responding on the previously active operand during the first day of EXT testing is commonly observed for most drug SA paradigms, and is thought to reflect goal-directed behavior (Peters et al., 2008; Bossert et al., 2012; Pittenger et al., 2017). As shown in Figure 3A, Tol SA rats showed a significant increase in ANPs during the first extinction trial as compared to the last week of toluene self-administration (Figure 3A, two-way repeated measures ANOVA session x nose poke interaction, *F*_(__14_,_140__)_ = 2.57, *p* = 0.0023; Sidak’s *post hoc*, EXT day 1 vs. SA *t*_(__140__)_ = 6.95, *p* < 0.0001). Air SA rats showed no increase in ANP responding during the first extinction trial (Figure 3B, Sidak’s *post hoc*, *t*_(__140__)_ = 0.97, *p* = 1.00). Tol SA, but not Air SA rats, also showed more ANPs relative to INPs on day 1 of extinction (Sidak’s *post hoc*, Tol SA, *t*_(__140__)_ = 4.94, *p* < 0.0001; Air SA, *t*_(__140__)_ = 1.08, *p* = 0.99).

During early EXT, Tol SA rats also evoked a greater number of would-be vapor deliveries as compared to Air SA rats (Figure 3B, two-way repeated measures ANOVA, treatment x day interaction, *F*_(__13_,_130__)_ = 5.24, *p* < 0.0001; main effect of treatment *F*_(__1_,_10__)_ = 54.30, *p* < 0.0001). This increase persisted for 4 days of EXT training (Sidak’s *post hoc*, Air vs. Tol, days 1–4, all *t*_(__140__)_ > 4.610 all *p* < 0.001) while over the final 2 weeks, responding of Tol SA rats was not different from that of Air SA rats and was significantly below the average Tol SA day (Sidak’s *post hoc*, Tol SA vs. EXT days 7–14, all *t*_(__140__)_ > 2.71, *p* < 0.05).

**FIGURE 4:**
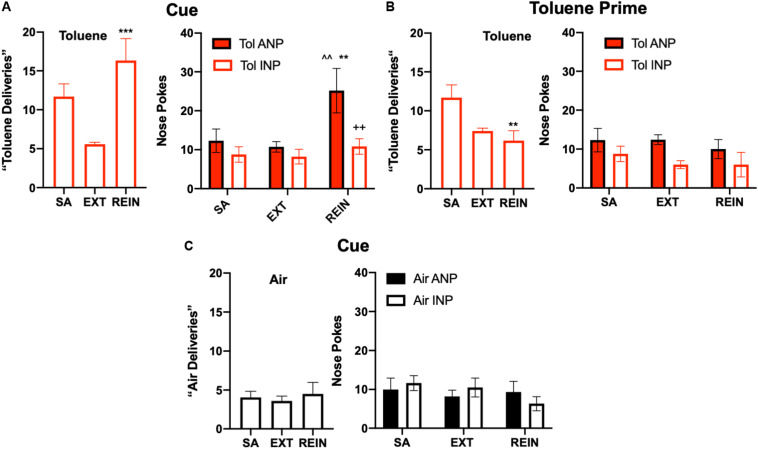
Reinstatement of toluene self-administration behavior. **(A)** Cue-induced reinstatement of responding in rats extinguished from toluene SA behavior. Left panel shows average vapor deliveries in rats self-administering toluene (SA; last week average), after extinction (EXT) or during re-instatement (REIN); ^∗∗∗^*p* < 0.001 REIN vs. EXT, Dunnett’s *post hoc*. Right panel shows vapor deliveries, active (ANP) and inactive nose pokes (INP; right panel) vapor deliveries in rats self-administering toluene (SA; last week average), after extinction (EXT) or during re-instatement (REIN); ^^*p* < 0.01 REIN vs. SA; ^∗∗^*p* < 0.01 REIN vs. EXT; ++*p* < 0.01 Tol ANP vs. Tol INP during REIN, Sidak’s *post hoc*. **(B)** A brief (15 s) exposure to 500 ppm toluene vapor prior to testing failed to reinstate responding in the active hole. One-way ANOVA and *post hoc* testing revealed a significant difference between SA and REIN groups (^∗∗^*p* < 0.01) Data shown are average vapor deliveries (left panel) or active (ANP) and inactive nose pokes (INP, right panel) in rats self-administering toluene (SA; last week average), after extinction (EXT) or during re-instatement (REIN). **(C)** Cues did not alter responding in Air rats. Data shown are average vapor deliveries (left panel) or active (ANP) and inactive nose pokes (INP; right panel) in rats self-administering Air (SA; last week average), after extinction (EXT) or during re-instatement (REIN). All data are mean ± SEM. Air *N* = 6, Tol *N* = 6.

Finally, cue- and drug-induced reinstatement (REIN) trials were conducted to measure drug-seeking behavior. Tol SA rats that had undergone extinction increased their number of toluene vapor deliveries (one-way repeated measures ANOVA, *F*_(__2_,_10__)_ = 11.66, *p* = 0.0024; Figure 4A, Left) as compared to EXT levels during cue-induced reinstatement (Dunnett’s *post hoc*, *t*_(__10__)_ = 4.81, *p* = 0.0013; Figure 4A, right). Two way ANOVA of nose poke (ANP, INP) and test session (SA, EXT, REIN) as factors revealed a significant main effect of test session (*F*_(__2_,_20__)_ = 6.35, *p* = 0.0073) in Tol SA rats (Figure 4A, Right). Sidak’s *post hoc* test revealed a greater number of ANPs than INPs specifically during cue-induced REIN (*t*_(__30__)_ = 3.35, *p* = 0.0067). There was a strong statistical trend for the nose poke x session interaction (*F*_(__2_,_20__)_ = 3.16, *p* = 0.064) and main effect of nose poke (*F*_(__1_,_10__)_ = 4.96, *p* = 0.05). As *post hoc* comparisons can be performed even if significance in the overall ANOVA is not found (Hsu, 1996; Maxwell et al., 2018), we compared ANPs across the various sessions. Sidak’s *post hoc* test revealed that ANPs during REIN were significantly greater than ANPs during SA (*t*_(__20__)_ = 3.49, *p* = 0.0069) and EXT (*t*_(__20__)_ = 3.92, *p* = 0.0026; Figure 4B). Unlike Tol SA rats, reintroduction of cues to Air SA animals did not cause reinstatement to air vapor responding (Figure 4C, Left; one-way repeated measures ANOVA, *F*_(__2_,_10__)_ = 0.41, *p* = 0.68) or nose pokes (Figure 4C, Right; nose poke x session interaction, *F*_(__2_,_20__)_ = 0.97, *p* = 0.40).

To test for drug-induced reinstatement, extinguished Tol SA rats were given a single exposure to 15 s of 500 ppm Tol immediately prior to testing. The number of toluene deliveries between SA, EXT, and REIN groups showed a significant difference upon analysis (Figure 4B, Left; one-way repeated measures ANOVA, *F*_(__2_,_10__)_ = 7.22, *p* = 0.012, Figure 4B, left) but this effect was due to a reduction in REIN responding compared to Tol SA (Sidak’s *post hoc* REIN vs. Tol SA, *t*_(__10__)_ = 3.62, *p* = 0.014). There was no difference in toluene deliveries between Tol EXT and Tol REIN (*t*_(__10__)_ = 0.81, *p* = 0.82) following the toluene prime. Further, there was no difference in nose pokes (Figure 4B, Right; nose poke × session interaction, two-way ANOVA *F*_(__2_,_20__)_ = 0.28, *p* = 0.76). Taken together, these results indicate that Tol SA rats did not reinstate drug-seeking in response to a drug prime.

## Discussion

### Using a Novel Self-Administration Model to Study the Reinforcing Properties of Toluene

In this study, we developed a novel method to test the feasibility of training rats to self-administer toluene vapor. Tol SA rats triggered an average of 11 vapor deliveries during daily 1 h SA sessions in the final week of testing, approximately twice that of Air SA rats. Although the total number of toluene deliveries per session was modest compared to that obtained with other addictive vapors including alcohol (de Guglielmo et al., 2017) and sufentanil (Vendruscolo et al., 2018), the findings of this study suggest that toluene appears to be acting as a reinforcer that drives goal-directed self-administration. This is supported by the finding that, during and following training, the majority of toluene exposures occurred in the beginning of a SA session, a common feature of drug SA paradigms (Ahmed and Koob, 1999; Griffin et al., 2009). While toluene vapor can induce significant motor impairment and sedation (Duncan et al., 2014), this is more often observed at concentrations and exposure times considerably greater than those used in our self-administration model. It is more likely that the reduction in responding that occurs over the self-administration session reflects a satiety or tolerance to further drug-induced reward. This could reflect changes in neural signaling induced by repeated toluene exposures that outlast the brief period of active vapor inhalation. Although not tested in the present study, we have previously shown that acute exposure of brain slices to toluene induces a long-lasting, endocannabinoid-dependent reduction in AMPA-mediated synaptic glutamatergic signaling in the medial prefrontal cortex (Beckley and Woodward, 2011) and nucleus accumbens (Beckley et al., 2016). Future studies could investigate whether manipulating endocannabinoid signaling alters the pattern of toluene self-administration. In addition to the temporal profile of toluene self-administration, the active lever preference, vapor delivery success rates, and sustained low latency to first delivery, are also all consistent with the idea that responding for toluene vapor may be driven by goal-directed behavior and not the result of a non-specific increase in nosepoke activity.

Following SA, rats underwent 3 weeks of extinction testing during which active nose pokes no longer triggered conditioned cues, discriminatory cues, or toluene deliveries. We observed a significant increase in the number of active nose pokes and would-be toluene deliveries on the first day of extinction training. This “extinction spike” is often observed in operant drug self-administration studies and is thought to reflect increased drug-seeking behavior during initial extinction trials during which animals expect to be rewarded (Peters et al., 2008; Bossert et al., 2012; Pittenger et al., 2017). Total vapor deliveries dropped below Tol SA levels by day 7 of responding and were indistinguishable from air extinction responding. This time frame is within range of other drug SA/extinction models (Peters et al., 2008; Bossert et al., 2012; Markou et al., 2018). Taken together, these findings are consistent with, albeit generally less robust than those reported for other drugs of abuse.

After extinction trials, rats underwent reinstatement testing where drug-seeking behavior can be triggered by the re-introduction of previously drug-paired cues or a non-contingent exposure to the drug (for review, see Venniro et al., 2016). Following 3 weeks of extinction training, Tol SA rats demonstrated cue- but not drug-induced reinstatement. The reason for the lack of toluene-induced reinstatement is not yet known but may indicate that vapor concentrations higher or lower than the 500 ppm exposure used in this study are required. This disparity may also be due to the fact that cue- and drug-primed reinstatement rely on overlapping, but not identical neurocircuitry. For instance, for psychostimulants, activation of nucleus accumbens shell-projecting infralimbic neurons (IL-NAcs) reduces cue- but not drug-primed reinstatement (Augur et al., 2016). Previous studies from our laboratory showed that exposure to toluene vapor reduces the intrinsic excitability of IL-NAcs neurons in a layer-dependent fashion (Wayman and Woodward, 2017) and that chemogenetic activation of the IL-NAcs pathway impairs the expression of toluene-induced conditioned place preference (Wayman and Woodward, 2018). Future studies could to test whether toluene self-administration produces similar effects on the excitability of NAc projecting mPFC neurons and if manipulating these neurons also disrupts cue-induced reinstatement to toluene.

### Methodological Considerations for Supporting Toluene Self-Administration in Rodents

To date, the majority of studies investigating toluene action have used passive methods of vapor exposure given the ease of use and control over exposure time. However, there are several critical advantages to using an inhalation-based model for toluene self-administration: (1) self-administration of toluene vapor has strong face-validity, as inhalation is the preferred route of administration in humans (2) drug intake is voluntary and thus, likely involves critical reward pathways that may be affected differently by non-contingent drug administration (Fernàndez-Castillo et al., 2012; Lominac et al., 2012). (3) longitudinal studies with repeated testing can be conducted over the course of the rodent’s lifetime (4) there are no concerns about catheter patency, that is especially important since toluene can cause vein damage (Kulkarni et al., 2015) and (5) no need for solubilizing agents (e.g., corn oil), commonly used when administering toluene intraperitoneally (Riegel et al., 2004; Lo et al., 2009; Lin et al., 2010; Chan et al., 2012; Wu et al., 2018).

Despite the general positive outcome of this initial toluene vapor self-administration study, there are several limitations that need to be addressed. First, the sample size was relatively small and reflects the low throughput achieved with the one-chamber system used in the current study. As the design of the toluene self-administration apparatus is modular and is easily expanded, future studies can use multiple chambers to allow for simultaneous testing of multiple animals as well as the use of yoked controls to explore differences between contingent and non-contingent exposures. Additional problems encountered during the testing of the system included obtaining dynamic control of vapor concentration, titrating the dose that would support self-administration, and attaining high vapor deliveries for a compound that depresses central nervous system activity. One method employed by Taffe and colleagues allows for dynamic control of vaporized nicotine, THC, and sufentanil using modified electronic cigarette technology (Vendruscolo et al., 2018; Javadi-Paydar et al., 2019; Nguyen et al., 2019). Unfortunately, this system would likely not be safe for generating toluene vapor since it relies on a superheated coil (100–250°C) to vaporize solutions and such heating of toluene might evoke combustion or uncontrolled changes in pressure. To bypass this concern, we placed a series of solenoid valves downstream of two sevoflurane vaporizers in order to control air flow and allow the rats to control the amount of toluene vapor via head entry into an “active” nose poke port, that triggered toluene vapor to the chamber. Results from the literature were used to determine an relevant dose of toluene delivered per vapor delivery. This is important variable since, like other CNS depressants, toluene has biphasic effects on locomotion (Evans and Balster, 1991) that could affect operant responding. While 560–1780 ppm toluene vapor increases locomotor activity in mice, the effect of slightly higher doses has been mixed (Kjellstrand et al., 1985; Wood and Colotla, 1990; Apawu et al., 2015). Toluene increases operant responding rates for food at 1000 to 1600 ppm toluene but decreases responding at 2000–3000 ppm in rodents (Glowa, 1981; Moser and Balster, 1981; Glowa et al., 1983). Further, concentrations from 1895 to 4950 ppm toluene cause conditioned place preference in rats (Gerasimov et al., 2003; Lee et al., 2004, 2006; Schiffer et al., 2006; Wayman and Woodward, 2018). Following preliminary testing with a range of vapor concentrations, we calibrated the system to generate a chamber concentration of ∼500 ppm toluene during a single 15 s vapor exposure. This resulted in an observed maximum concentrations of toluene vapor of ∼2000 ppm in rodents self-administering toluene multiple times over a short period of time. Thus, rats in the present study self-administered a dose of toluene vapor shown to cause conditioned place preference but one that is below the range associated with overt sedation or task disengagement. Future studies can test additional dose ranges to optimize self-administration behavior.

In addition to dosing, several features were found that helped generate fairly stable levels of responding for toluene vapor. The first 2 weeks of non-contingent training provided critical habituation to potential stressors that might otherwise limit or impair acquisition of operant responding. Namely, daily exposures to a low level (100 ppm) of toluene vapor in the operant box allowed acclimation to toluene odor while minimizing intoxicating effects (Weiss et al., 1979; Funada et al., 2002; Gerasimov et al., 2003; Lee et al., 2006). During this time, rodents also habituated to the sound of the solenoid values that are triggered by entry into the active nose poke. An additional critical aspect of the design was the location of the delivery of toluene vapor to the chamber following an active response. Initial attempts to induce self-administration used a brief delivery of toluene vapor directly into the nose poke hole. This was unsuccessful as during initial trials, rodents typically retreated from the nose poke thus limiting exposure to rewarding concentrations of toluene. Recent studies also suggest that non-contingent drug inhalation prior to or in combination with self-administration training promotes drug-seeking across other several drugs of abuse including THC (Spencer et al., 2018), nicotine (Gilpin et al., 2014), and alcohol (Roberts et al., 2000; Rimondini et al., 2002; Becker and Lopez, 2004). Passive exposures can enhance the rewarding properties of these drugs (including toluene), that might aid the acquisition of self-administration behavior (Fidler et al., 2011; Wayman and Woodward, 2017; Spencer et al., 2018; Javadi-Paydar et al., 2019; Ponzoni et al., 2019). Alternatively, these exposures might precipitate withdrawal, causing increased drug self-administration as a means to alleviate negative symptoms (George et al., 2010; Heilig et al., 2010; Ponzoni et al., 2015). Regardless, omission of these pre-exposure steps yielded low operant responding in rats self-administering toluene vapor (unpublished observations). That said, it is noted that such passive exposures may not accurately model the ways in which humans acquire solvent self-administration although it is likely that, similar to other drugs of abuse, they may engage in exploratory exposures that predispose future use.

Future studies specifically geared toward modeling escalated drug use could consider increasing the 1 h duration of daily toluene SA sessions used in this study. For many drugs, long-access (6 + h) models have been shown to enhance intake better than short-access models (Ahmed and Koob, 1999; Picetti et al., 2012; Vendruscolo et al., 2018). However extended access studies will need to consider the potential drawbacks of long periods of toluene exposure such as brain-wide oxidative stress (Kodavanti et al., 2015), impaired visual function that may inhibit cue recognition (Boyes et al., 2016) pulmonary inflammation (Kanter, 2011) and locomotor depression that may diminish the ability of the animal to administer the toluene vapor. Finally, studies where active response preference is the primary dependent variable (such as testing reinstatement-mitigating treatments) could consider increasing the fixed-ratio for drug responding in order to increase active responding.

## Conclusion

We developed a novel, low-cost, scalable protocol by which rodents learned to self-administer toluene vapor and reinstate responding to previously extinguished cues. This protocol should be useful in studies seeking to uncover the cellular, molecular, or physiological bases of inhalant addiction, as well as testing compounds with potential for inhalant relapse prevention.

## Data Availability Statement

The datasets generated for the figures shown in this study are available upon request to the corresponding author.

## Ethics Statement

The animal study was reviewed and approved by the Medical University of South Carolina Institutional Animal Care and Use Committee.

## Author Contributions

KB, WW, and JW designed and built the self-administration apparatus, performed the data analysis, and wrote the manuscript. KB, WW, and MO conducted the experiments. All authors contributed to the article and approved the submitted version.

## Conflict of Interest

The authors declare that the research was conducted in the absence of any commercial or financial relationships that could be construed as a potential conflict of interest.
